# Prevalence and Variation of Developmental Screening and Surveillance in Early Childhood

**DOI:** 10.1001/jamapediatrics.2018.1524

**Published:** 2018-07-09

**Authors:** Ashley H. Hirai, Michael D. Kogan, Veni Kandasamy, Colleen Reuland, Christina Bethell

**Affiliations:** 1 Maternal and Child Health Bureau, Health Resources and Services Administration, Rockville, Maryland; 2 Oak Ridge Institute for Science and Education, Oak Ridge, Tennessee; 3 Department of Pediatrics, Oregon Health and Sciences University, Portland; 4 Department of Population, Family, and Reproductive Health, Johns Hopkins Bloomberg School of Public Health, Baltimore, Maryland

## Abstract

**Importance:**

Since 2001, the American Academy of Pediatrics has recommended universal developmental screening and surveillance to promote early diagnosis and intervention and to improve the outcomes of children with developmental delays and disabilities.

**Objective:**

To examine the current prevalence and variation of developmental screening and surveillance of children by various sociodemographic, enabling, and health characteristics.

**Design, Setting, and Participants:**

This cross-sectional analysis of the Health Resources and Services Administration’s 2016 National Survey of Children’s Health—a nationally representative survey of US children completed between June 2016 and February 2017—examined 5668 randomly selected children 9 through 35 months of age whose parent or caregiver responded to the address-based survey by mail or via a website. All analyses were weighted to account for the probability of selection and nonresponse and to reflect population counts of all noninstitutionalized US children residing in housing units.

**Main Outcomes and Measures:**

Developmental screening was measured through a validated set of 3 items indicating receipt in the past year of parent-completed screening from a health care professional with age-appropriate content regarding language development and social behavior. Surveillance was determined by an item capturing verbal elicitation of developmental concerns by a health care professional.

**Results:**

Of the estimated 9.0 million children aged 9 through 35 months, an estimated 30.4% (95% CI, 28.0%-33.0%) were reported by their parent or guardian to have received a parent-completed developmental screening and 37.1% (95% CI, 34.4%-39.8%) were reported to have received developmental surveillance from a health care professional in the past year. Characteristics associated with screening and/or surveillance that remained significant after adjustment included primary household language, family structure, household education, income, medical home, past-year preventive visit, child health status, and special health care needs. Having health care that meets medical home criteria was significantly associated with both developmental screening (adjusted rate ratio, 1.34; 95% CI, 1.13-1.57) and surveillance (adjusted rate ratio, 1.24; 95% CI, 1.08-1.42), representing an 8 to 9 absolute percentage point increase. State-level differences spanned 40 percentage points for screening (17.2% in Mississippi and 58.8% in Oregon) and surveillance (19.1% in Mississippi and 60.8% in Oregon), with approximately 90% of variation not explained by child and family characteristics.

**Conclusions and Relevance:**

Despite more than a decade of initiatives, rates of developmental screening and surveillance remain low. However, state-level variation indicates continued potential for improvement. Systems-level quality improvement efforts, building on the medical home, will be necessary to achieve recommended screening and surveillance goals.

## Key Points

**Question:**

What are the latest national estimates of standardized developmental screening and surveillance, as well as individual and state variation, that may identify opportunities for improvement?

**Findings:**

In this cross-sectional analysis of the 2016 National Survey of Children’s Health, an estimated 30.4% of children 9 through 35 months of age received a parent-completed developmental screening and 37.1% received developmental surveillance from a health care professional in the past year. State-level differences far exceeded those by child and family characteristics, spanning 40 percentage points for screening (17.2% in Mississippi and 58.8% in Oregon) and surveillance (19.1% in Mississippi and 60.8% in Oregon).

**Meaning:**

Overall rates of developmental screening and surveillance remain low; however, substantial state-level variation underscores the importance and potential of quality improvement efforts.

## Teaser

This cross-sectional analysis uses the 2016 National Survey of Children’s Health to examine the current prevalence and variation of developmental screening and surveillance of children by various sociodemographic, enabling, and health characteristics.

## Introduction

Approximately 12% to 15% of US children experience developmental delays or disabilities, which range in severity and scope from isolated delays in achieving certain developmental milestones to functional impairments in hearing or vision, as well as diagnosable learning, emotional, and behavioral disorders.^1,2^ Early identification and intervention are critical to optimize language, cognitive, motor, and socioemotional development as well as educational success,^3,4,5^ yet only an estimated 10% of children with delays are identified and receive intervention.^2^ The American Academy of Pediatrics (AAP) first recommended developmental screening in 2001 and later expanded guidelines in 2006 with an algorithm that included standardized developmental screening at 9-, 18-, and 30-month well-child visits or whenever a parent or clinician expresses concern through ongoing surveillance at every preventive visit, as well as autism-specific screening at 18 and 24 months of age.^6,7,8^ Subsequent randomized clinical trials of standardized developmental screening relative to surveillance alone and interventions to increase screening have bolstered these recommendations by demonstrating improvements in timely diagnosis, treatment referrals, and early intervention.^9,10^

During the past decade, there have been several initiatives to improve developmental screening, including the AAP’s Developmental Surveillance and Screening Policy Implementation Project in 17 pediatric practices,^11^ Pediatric Improvement Partnerships in 12 states and Washington, DC,^12,13^ and multiple iterations of the Commonwealth Fund’s Assuring Better Child Development initiative, which supported 26 states, Washington, DC, and Puerto Rico to implement policy and practice changes.^14^ Federal campaigns have included the Agency for Children and Family’s “Birth to Five, Watch me Thrive” program and the Centers for Disease Control and Prevention’s “Learn the Signs. Act Early” program. Developmental screening was included as a Healthy People 2020 objective,^15^ as part of the Medicaid and Children’s Health Insurance Program child core set of quality measures with demonstration grants for 10 states,^16^ and, most recently, as a national performance measure for the Title V Maternal and Child Health Services Block Grant,^17^ with 42 states electing to work toward improvement during the 5-year planning cycle beginning in 2015.

Data from the National Survey of Children’s Health (NSCH), the only nationally representative data source for standardized developmental screening, indicate that, in 2007, fewer than 1 in 5 children had received a standardized parent-completed developmental screening from a health care professional in the past year,^18,19^ which increased to about 1 in 3 children by 2011-2012.^20,21^ Substantial state-level variation, both within and across survey years, far exceeded differences by child or household characteristics. Using the 2016 NSCH, we sought to provide a current examination of the prevalence and variation of recommended developmental screening and surveillance, which serves to establish a baseline for new initiatives and identify opportunities for improvement.

## Methods

### Data Source and Study Population 

Data for this analysis come from the 2016 NSCH—a nationally representative, parent-completed survey of US children younger than 18 years funded and directed by the Health Resources and Services Administration and conducted by the US Census Bureau. Relative to prior survey iterations in 2003, 2007, and 2011-2012, the 2016 NSCH was significantly redesigned to merge content with the former National Survey of Children With Special Health Care Needs and, owing to declining response rates, to change administration from telephone-based interviews to a mailed, self-administered survey with paper and web-based response options. Therefore, data from the 2016 NSCH are not directly comparable to data from prior years of the NSCH. After a parent or caregiver completed a household-based screening instrument to determine the presence of children by special health care needs status, 1 child per household was selected for the survey, with oversampling for those with special health care needs. Surveys were available in both English and Spanish and were completed between June 2016 and February 2017 (N = 50 212). The survey completion rate among households with a confirmed child was 69.7%, and the overall response rate including households without children was 40.7%. All analyses were weighted to account for the unequal probability of selection and differential nonresponse by various sociodemographic factors, and represent all noninstitutionalized US children residing in housing units. Additional details of the NSCH are available elsewhere.^22,23^ Participation was voluntary and confidential under Title 13, United States Code, Section 9. Prior to public release, all data products are reviewed for adherence to privacy protection and disclosure avoidance guidelines by the US Census Bureau’s Disclosure Review Board.

We restricted the study population to children between the ages of 9 and 35 months (N = 5668) for consistency with AAP guidelines for developmental screening^7^; Healthy People 2020 Maternal, Infant, and Child Health Objective 29.1^15^; the Medicaid and Children’s Health Insurance Program core quality measure^24^; and other initiatives aimed at children from birth to 3 years of age.

### Outcomes

#### Developmental Screening

The NSCH captures the receipt of standardized, parent-completed developmental screening, hereafter referred to as *developmental screening*, through 3 survey items that were iteratively developed and validated.^18^ Children are considered to have received developmental screening if a parent or caregiver responded affirmatively to whether a doctor or health care professional had them or another caregiver complete a questionnaire about specific concerns or observations they may have had about the child’s development, communication, or social behavior in the past year and whether this questionnaire included 2 additional age-specific content components capturing language development and social behavior. Although not all standardized screening tools involve a parent-completed component, they are more commonly used by pediatricians and are favored for both their efficiency and their ability to engage parents.^11,25^ A total of 5492 children 9 through 35 months of age (96.9%) had complete parental or caregiver responses to the developmental screening survey items.

#### Developmental Surveillance

Children were considered to have received developmental monitoring or surveillance by a health care professional if a parent or caregiver responded affirmatively to 1 item assessing whether physicians or other health care professionals asked if they had concerns about their child’s learning, development, or behavior. This measure captures the first of 5 recommended surveillance steps: eliciting parental concerns about their child’s development, documenting and maintaining a developmental history, making accurate observations of the child, identifying risk and protective factors, and maintaining an accurate record of documenting the process and findings.^7^ Although elicitation may occur through a previsit questionnaire similar to a parent-completed screening tool, this surveillance measure captures complementary verbal communication regarding developmental concerns. A total of 5652 children 9 through 35 months of age (99.7%) had a valid parental or caregiver response to the developmental surveillance survey item.

### Covariates

Following the behavioral model of health care use,^26,27,28,29^ various child-, family-, and health care–related factors were examined that may influence developmental screening and surveillance through parent and clinician behavior. Predisposing sociodemographic characteristics that may influence the use or receipt of services included the child’s age, sex, race/ethnicity, primary language, family structure, and highest household educational level. Enabling factors that may influence the ability to access developmental screening included household income to poverty ratio, insurance coverage and type, the presence of a medical home, having a preventive visit in the past year, and state of residence. Health status characteristics that may influence a perceived need for screening by parents or health care professionals included the child’s health status and the presence of a special health care need. The NSCH measures the AAP-defined medical home through a multi-item composite of 5 components: having a usual source of care, having a personal physician or nurse, receiving family-centered care, receiving referrals for specialty care if needed, and receiving help coordinating health and health-related care if needed.^30^ Special health care need status is determined using a validated screener that captures chronic physical, developmental, behavioral, or emotional conditions requiring increased use of health or related services.^31,32^ Owing to a large percentage of missing data (18.6%) and use in the weighting process, the household income to poverty ratio was multiply imputed by the US Census Bureau using regression imputation methods.^22^

### Statistical Analysis

Bivariate associations between the covariates and the receipt of screening and surveillance were examined using χ^2^ tests for significance. Multivariable logistic regression models were used to estimate adjusted associations with screening and surveillance using all covariates in a single model. State-level estimates were achieved with fixed effects and complex variance estimation to account for clustering within states. To improve interpretation and translation, estimated odds were converted to marginal probabilities for presentation of adjusted prevalence estimates and rate ratios.^33^ Unadjusted and model-adjusted state-level prevalence estimates were compared to assess the contribution of child-, family-, and health care–related characteristics in explaining state variation. State-level estimates were also compared with national estimates using *t* tests for overlapping groups.^34^ Given the potential mediating role of preventive services and the possibility that certain special health care needs are identified through developmental screening, sensitivity analyses were conducted without these covariates in multivariable models. Approximately 5% of the study sample had missing data on nonimputed covariates and were excluded from regression analyses. All analyses adjusted the variance estimates to account for the complex survey design and multiple imputation of poverty using SAS-callable SUDAAN, version 11.0.1 (Research Triangle Institute).

## Results

In 2016, an estimated 30.4% (95% CI, 28.0%-33.0%) of children 9 through 35 months of age were reported by a parent or caregiver to have received a parent-completed developmental screening from a health care professional in the past year that included assessment of communication and behavior (Table 1). A higher percentage of children (37.1%; 95% CI, 34.4%-39.8%) had received developmental surveillance in which a health care professional had asked if the parent or caregiver had any concerns about their development. Slightly less than 1 in 5 children (19.2%) had received both screening and surveillance, while more than half (51.6%) had received neither screening nor surveillance (Table 2).

**Table 1.** Developmental Screening and Surveillance by Predisposing, Enabling, and Need Characteristics

Characteristic	Overall Distribution (N = 5668), Weighted %	Developmental Screening (n = 5492)		Developmental Surveillance (n = 5652)	
		Weighted % (95% CI)	*P* Value^a^	Weighted % (95% CI)	*P* Value^a^
Total	100.0	30.4 (28.0-33.0)		37.1 (34.4-39.8)	
Weighted population size, No.	9.0 million	2.7 million		3.3 million	
**Predisposing Characteristics**					
Age, mo					
9-23	55.5	29.4 (26.0-32.9)	.33	36.1 (32.4-40.1)	.66
24-35	44.5	31.8 (28.4-35.3)		38.2 (34.7-41.9)	
Sex					
Male	51.5	32.0 (28.4-35.7)	.21	38.7 (35.0-42.6)	.22
Female	48.5	28.8 (25.7-32.1)		35.3 (31.7-39.2)	
Race/ethnicity					
Non-Hispanic white	53.1	34.4 (31.7-37.1)	<.001	40.1 (37.3-43.0)	.03
Non-Hispanic black	11.9	24.8 (16.7-35.2)		30.4 (22.7-39.5)	
Hispanic	22.5	24.3 (18.5-31.3)		32.6 (25.5-40.8)	
Non-Hispanic other single race	6.2	20.2 (14.1-28.1)		31.2 (23.1-40.7)	
Non-Hispanic multiple race	6.3	38.8 (30.3-48.2)		45.3 (36.5-54.5)	
Primary household language					
English	84.7	33.3 (30.8-35.9)	<.001	38.5 (35.9-41.2)	.02
Non-English	15.3	14.2 (9.6-20.4)		26.4 (18.4-36.3)	
Family structure					
2 Parents, married	67.8	34.8 (31.9-37.8)	<.001	39.9 (37.0-42.9)	.008
2 Parents, unmarried	12.6	25.7 (18.9-33.9)		35.3 (27.2-44.4)	
Single mother or other	19.6	20.9 (16.1-26.6)		28.6 (22.8-35.2)	
Highest household educational level					
<High school	7.3	16.0 (7.6-30.6)	<.001	20.3 (10.7-35.1)	.03
High school	17.4	22.4 (16.5-29.7)		34.1 (26.9-42.1)	
Some college	22.2	25.8 (21.4-30.7)		37.6 (32.1-43.5)	
≥College degree	53.1	37.7 (34.7-40.9)		40.5 (37.6-43.5)	
**Enabling Characteristics**					
Household income-to-poverty ratio, % federal poverty level					
<100	21.3	22.7 (16.6-30.2)	.009	32.1 (25.0-40.1)	.24
100-199	20.7	26.1 (20.6-32.4)		40.9 (33.6-48.7)	
200-399	28.8	33.9 (29.2-39.0)		35.5 (30.6-40.9)	
≥400	29.3	36.1 (32.3-40.0)		39.4 (35.9-43.1)	
Insurance coverage and type					
Any public	36.8	23.7 (19.8-28.1)	<.001	36.5 (31.5-41.7)	.04
Private only	58.2	36.0 (32.9-39.1)		39.0 (36.0-42.2)	
Uninsured	5.0	16.9 (8.7-30.3)		20.1 (10.1-36.0)	
Has medical home					
Yes	53.0	37.1 (33.9-40.5)	<.001	41.8 (38.4-45.1)	<.001
No	47.0	22.7 (19.3-26.5)		31.7 (27.7-36.0)	
Preventive visit in past year					
Yes	91.1	32.3 (29.8-35.0)	<.001	39.5 (36.8-42.4)	<.001
No	8.9	9.2 (5.7-14.7)		13.1 (7.6-21.7)	
**Need Characteristics**					
Child health status					
Excellent or very good	93.8	31.4 (28.9-34.1)	.006	37.0 (34.3-39.8)	.60
Good, fair, or poor	6.2	17.7 (11.6-26.1)		40.2 (28.9-52.7)	
Special health care needs status					
Yes	8.0	39.1 (31.4-47.4)	.02	53.3 (44.8-61.6)	<.001
No	92.0	29.7 (27.2-32.3)		35.6 (32.9-38.5)	

^a^ Determined by the χ^2^ test.

**Table 2.** Developmental Screening by Surveillance

Developmental Screening (n = 5481)	Developmental Surveillance, %	
	Yes	No
Yes	19.2	11.3
No	17.9	51.6

Several sociodemographic characteristics were associated with both screening and surveillance (Table 1). For example, non-Hispanic white children were about 10 percentage points more likely than non-Hispanic black or Hispanic children to have received screening (non-Hispanic white, 34.4%; 95% CI, 31.7%-37.1% vs non-Hispanic black 24.8%; 95% CI, 16.7%-35.2% and Hispanic, 24.3%; 95% CI, 18.5%-31.3%) and surveillance (non-Hispanic white, 40.1%; 95% CI, 37.3%-43.0% vs non-Hispanic black, 30.4%; 95% CI, 22.7%-39.5% and Hispanic, 32.6%; 95% CI, 25.5%-40.8%). There was also a strong educational gradient, with children of college-educated parents at least 20 percentage points more likely than those with less than a high school degree to have received screening (college educated, 37.7%; 95% CI, 34.7%-40.9%; less than high school degree, 16.0%; 95% CI, 7.6%-30.6%) and surveillance (college educated, 40.5%; 95% CI, 37.6%-43.5%; less than high school degree, 20.3%; 95% CI, 10.7%-35.1%). Other enabling and health factors associated with both screening and surveillance included having a medical home (screening, 37.1%; 95% CI, 33.9%-40.5% vs 22.7%; 95% CI, 19.3%-26.5%; surveillance, 41.8%; 95% CI, 38.4%-45.1% vs 31.7%; 95% CI, 27.7%-36.0%), a past-year preventive visit (screening, 32.3%; 95% CI, 29.8%-35.0% vs 9.2%; 95% CI, 5.7%-14.7%; surveillance, 39.5%; 95% CI, 36.8%-42.4% vs 13.1%; 95% CI, 7.6%-21.7%), and a special health care need (screening, 39.1%; 95% CI, 31.4%-47.4% vs 29.7%; 95% CI, 27.2%-32.3%; surveillance, 53.3%; 95% CI, 44.8%-61.6% vs 35.6%; 95% CI, 32.9%-38.5%). However, several other factors were associated with screening only, such as income (≥400% poverty, 36.1%; 95% CI, 32.3%-40.0% vs <100% poverty, 22.7%; 95% CI, 16.6%-30.2%) and insurance type (private only, 36.0%; 95% CI, 32.9%-39.1% vs any public, 23.7%; 95% CI, 19.8%-28.1%).

After adjustment with logistic regression models, most factors except for race/ethnicity and insurance remained significantly associated with screening or surveillance (Table 3). Similar to bivariate associations, there were more factors associated with standardized screening than with more general surveillance. Specifically, primary household language, highest household educational level, and child health status were only associated with screening. Compared with children living in an English primary language household, those in non-English primary language households were 40% less likely to have received screening in the past year (adjusted rate ratio, 0.60; 95% CI, 0.39-0.92). Having a medical home was associated with a 34% increase in screening (adjusted rate ratio, 1.34; 95% CI, 1.13-1.57) and a 24% increase in surveillance (adjusted rate ratio, 1.24; 95% CI, 1.08-1.42), corresponding to an 8 to 9 absolute percentage point increase. In additional models, medical home components—usual source of care, personal physician or nurse, and family-centered care—were associated with both screening and surveillance (eTable 1 in the Supplement). The small proportion of children without a past-year preventive visit were approximately 60% less likely to have received screening and surveillance, while those with special health care needs were more than 50% more likely to have received screening and surveillance. Additional models without a past-year preventive visit and special health care need status did not substantively alter associations (eTable 2 in the Supplement).

**Table 3.** Adjusted Associations With Developmental Screening and Surveillance

Characteristic	Developmental Screening (n = 5229)		Developmental Surveillance (n = 5373)	
	Adjusted Prevalence	Rate Ratio (95% CI)	Adjusted Prevalence	Rate Ratio (95% CI)
**Predisposing Characteristics**				
Age, mo				
9-23	30.2	1 [Reference]	36.4	1 [Reference]
24-35	32.7	1.08 (0.94-1.25)	37.7	1.03 (0.91-1.17)
Sex				
Male	32.7	1 [Reference]	38.3	1 [Reference]
Female	29.9	0.92 (0.80-1.05)	35.7	0.93 (0.82-1.06)
Race/ethnicity				
Non-Hispanic white	31.0	1 [Reference]	36.7	1 [Reference]
Non-Hispanic black	31.3	1.01 (0.72-1.42)	33.5	0.91 (0.69-1.21)
Hispanic	32.7	1.06 (0.84-1.34)	37.9	1.03 (0.83-1.28)
Non-Hispanic other single race	25.4	0.82 (0.58-1.16)	37.4	1.02 (0.76-1.37)
Non-Hispanic multiple race	36.0	1.16 (0.91-1.49)	43.2	1.18 (0.96-1.45)
Primary household language				
English	32.9	1 [Reference]	38.2	1 [Reference]
Non-English	19.8	0.60 (0.39-0.92)	28.7	0.75 (0.52-1.08)
Family structure				
2 Parents, married	33.4	1 [Reference]	38.9	1 [Reference]
2 Parents, unmarried	30.2	0.90 (0.68-1.21)	36.1	0.93 (0.72-1.19)
Single mother or other	24.0	0.72 (0.54-0.96)	30.4	0.78 (0.62-0.99)
Highest household educational level				
<High school	29.5	0.85 (0.47-1.56)	28.7	0.73 (0.41-1.29)
High school	26.5	0.77 (0.58-1.02)	32.5	0.83 (0.65-1.05)
Some college	26.6	0.77 (0.62-0.95)	36.4	0.92 (0.78-1.10)
≥College degree	34.5	1 [Reference]	39.3	1 [Reference]
**Enabling Characteristics**				
Household income-to-poverty ratio, % federal poverty level				
<100	34.4	1.20 (0.82-1.76)	37.9	1.09 (0.84-1.41)
100-199	31.2	1.09 (0.82-1.46)	43.6	1.26 (1.02-1.55)
200-399	33.0	1.15 (0.95-1.39)	34.5	0.99 (0.84-1.17)
≥400	28.7	1 [Reference]	34.7	1 [Reference]
Insurance coverage and type				
Any public	30.1	0.93 (0.74-1.18)	39.6	1.11 (0.90-1.36)
Private only	32.2	1 [Reference]	35.8	1 [Reference]
Uninsured	26.6	0.83 (0.49-1.39)	33.9	0.95 (0.55-1.63)
Has medical home				
Yes	35.1	1.34 (1.13-1.57)	40.4	1.24 (1.08-1.42)
No	26.3	1 [Reference]	32.6	1 [Reference]
Preventive visit in past year				
Yes	32.6	1 [Reference]	38.6	1 [Reference]
No	12.3	0.38 (0.23-0.62)	16.8	0.43 (0.27-0.70)
**Need Characteristics**				
Child health status				
Excellent or very good	32.1	1 [Reference]	36.8	1 [Reference]
Good, fair, or poor	18.8	0.58 (0.41-0.84)	39.3	1.07 (0.83-1.37)
Special health care needs status				
Yes	46.7	1.55 (1.29-1.85)	54.5	1.54 (1.31-1.80)
No	30.2	1 [Reference]	35.5	1 [Reference]

**Figure 1.**
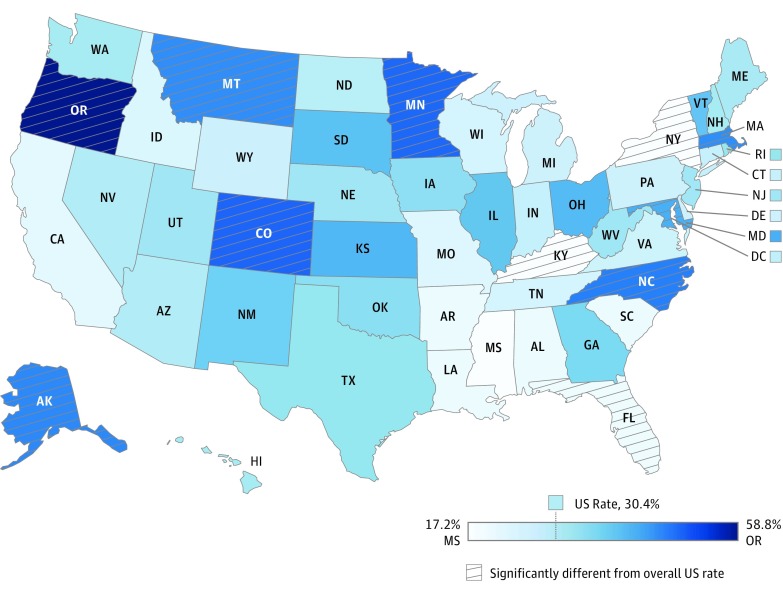
Developmental Screening Rates by State—National Survey of Children’s Health, 2016 This map illustrates state rates of developmental screening in a continuous blue color, ranging from 17.2% in Mississippi (lightest blue) to 58.8% in Oregon (darkest blue).

**Figure 2.**
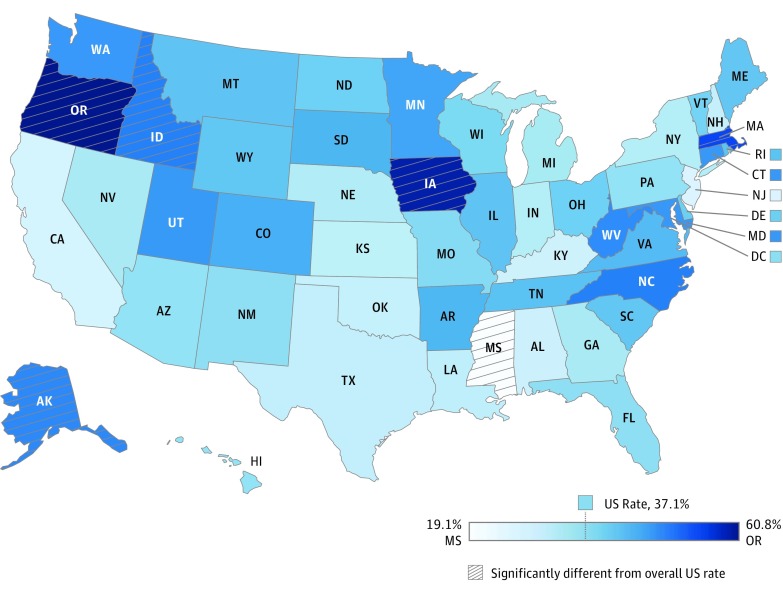
Developmental Surveillance Rates by State—National Survey of Children’s Health, 2016 This map illustrates state rates of developmental surveillance in a continuous blue color, ranging from 19.1% in Mississippi (lightest blue) to 60.8% in Oregon (darkest blue).

Both screening and surveillance varied substantially across states by more than 40 percentage points (Figure 1 and Figure 2). The prevalence of screening ranged from 17.2% in Mississippi to 58.8% in Oregon, corresponding to a rate ratio of 3.4. Similarly, developmental surveillance ranged from 19.1% in Mississippi to 60.8% in Oregon, corresponding to a rate ratio of 3.2. States with significantly lower rates of developmental screening than the nation overall included Kentucky (17.5%), New York (17.5%), and Florida (20.4%), while states with rates significantly exceeding the national rate included Oregon (58.8%), Colorado (50.2%), Minnesota (50.1%), North Carolina (47.6%), Alaska (46.8%), Montana (46.3%), Massachusetts (46.3%), and Maryland (43.0%). Mississippi was the only state with a significantly lower surveillance rate (19.1%) than the nation overall, while Oregon (60.8%), Iowa (59.4%), Massachusetts (54.9%), Alaska (53.9%), and Idaho (49.8%) had significantly higher rates. The correlation coefficient between state-level screening and surveillance rates indicated only a moderate association (*r* = 0.55), with 4 states having a 20-percentage point difference between surveillance and screening. Adjustment for sociodemographic, health care, and health status characteristics explained only 4% and 13% of the state-level variance in screening and surveillance, respectively, with a mean absolute state change of 1 percentage point (eTables 3 and 4 in the Supplement).

## Discussion

The results of this analysis indicate that less than one-third of all children 9 through 35 months of age have received a standardized parent-completed developmental screening from a health care professional in the last year. At 37.1%, developmental surveillance or elicitation regarding concerns during a health care visit is not substantially higher, and only 1 in 5 children received both screening and surveillance. Although ongoing surveillance is recommended to initiate formal screening for those at risk for delay, universal screening is shown to be more effective than surveillance alone at identifying delays and receiving referrals and intervention eligibility on a timelier basis.^10^ Thus, the low rates of screening are concerning and were no higher than 39.1% by various child, family, and health care characteristics.

In bivariate analyses, children of minority race/ethnicity, single mothers, and less-educated or lower-income parents, who were either uninsured or publicly insured, tended to have lower levels of screening and/or surveillance than their corresponding counterparts. This finding represents a notable shift from 2007, when these less-advantaged groups were more likely to be screened,^18,19,35^ perhaps as a result of a perceived risk of delay and/or efforts that focused on poor children, such as the Early and Periodic Screening Diagnostic and Treatment and Assuring Better Child Development programs.^14^ As rates of screening and general awareness of its importance have increased, familiar patterns of sociodemographic advantage in accessing quality care may have emerged.

Even after adjustment for other characteristics, children living in non–English-speaking primary language households were considerably less likely to receive screening, which demonstrates the continued relevance of previously identified language barriers,^18,19,36^ despite the availability of parent-completed screening tools in at least 14 languages.^7^ This disparity may also reflect issues of cultural competence and health care quality, given that only the rates of screening and not surveillance were significantly lower for children living in non–English-speaking primary language households. That fewer factors were significantly associated with surveillance in general may reflect the greater ease with which simple monitoring can occur owing to frequently cited clinician screening barriers of time and staffing limitations and inadequate reimbursement.^36,37^

Early identification of developmental disorders is an explicit function of the medical home,^38^ which is among the more modifiable enabling characteristics to increase rates of screening and surveillance. Previous studies have shown an unadjusted association between the medical home and developmental screening^19^ and an adjusted association with developmental surveillance.^29^ To our knowledge, this is the first study to have examined and reported an adjusted association between the medical home and rates of developmental screening, with 3 of 5 components driving this association: having a usual source of care, having a personal physician or nurse, and receiving family-centered care. Thus, efforts to promote continuous and comprehensive primary care within a medical home may result in improved quality and use of preventive services,^39^ including developmental screening and surveillance. The potential for significant improvement exists, with nearly half of children 9 to 35 months of age lacking a medical home, as estimated in this study.

Financial barriers to preventive services, including developmental screening, have been nearly eliminated through expansions of coverage and benefit requirements for private plans to cover all Bright Futures recommendations. Early and Periodic Screening Diagnostic and Treatment requires the same for state Medicaid programs, although details of coverage, codes, and rates of reimbursement may need to be updated in guidelines.^40^ More than 90% of young children were reported to have had a preventive visit in the past year, consistent with data from other surveys.^41,42^ However, other data suggest that there are still gaps in children receiving the recommended number of well-child visits,^42,43^ which could be addressed through reminder-recall and home visiting programs.^44,45^ Nonetheless, clinician-focused efforts including training, automated prompts in electronic medical records, and learning collaboratives may be necessary and are shown to be effective in promoting adherence to screening recommendations.^9,46^

Similar to previous analyses,^18,20^ state variation far exceeded that observed by child and family characteristics, with a range of more than 40 percentage points on an absolute scale and a tripling on a relative scale. Moreover, most of this variation could not be explained by available sociodemographic, enabling, or health characteristics, which suggests a role for unmeasured policies, practices, and quality improvement efforts. That many top performers, including Alaska, Colorado, Maryland, Massachusetts, Montana, and Oregon, have likely more than doubled their rates of screening in the past decade demonstrates that improvement is possible across the country. In particular, Oregon had one of the lowest rates of developmental screening in 2007^18^ and now has the highest rate in the nation—nearly twice the national rate, at 58.5%. This success may be attributable to tracking and incentivizing quality improvement through pay-for-performance metrics in coordinated care organizations, established as part of a Medicaid demonstration waiver. The medical home and developmental screening are among 5 emphasized incentive metrics, the latter of which tripled from 20.9% in 2011 to 62.2% in 2016.^47^

### Limitations

The major limitation of this analysis involves potential underestimation of developmental screening by capturing only screenings with a parent-completed component. However, physicians favor parent-completed screening tools for their efficiency and ability to engage parents in the process of developmental promotion.^11,25^ Our estimate of developmental screening is roughly comparable to the median of the 2016 Medicaid claims-based reporting from 26 states (36%) that includes tools without a parent-completed component.^48^ It is lower than the latest available AAP member survey report of always or almost always using any formal screening tool (47.7% in 2009)^25^; however, pediatricians may overreport screening and not all children receive primary care from AAP member pediatricians. Conversely, surveillance may be overestimated given that only the first of 5 recommended surveillance steps is captured. Regardless of counterbalancing assessment issues, these data represent the only current national estimates and indicate that only half of children younger than 3 years have received either screening or surveillance.

## Conclusions

Despite more than a decade of initiatives, the rates of developmental screening and surveillance remain low. However, state-level variation indicates continued potential for improvement. Systems-level quality improvement efforts that integrate the medical home and build on lessons learned from state-based initiatives will be necessary to achieve universal screening and surveillance that optimizes early identification, intervention, and developmental trajectories for children with delays.

## Notes

Supplement. **eTable 1.** Adjusted Associations Between Medical Home Components and Developmental Screening and Surveillance **eTable 2.** Adjusted Associations With Developmental Screening and Surveillance (Without Preventive Medical Visit and Special Health Care Needs Status) **eTable 3.** Unadjusted and Adjusted Developmental Screening Rates by State **eTable 4.** Unadjusted and Adjusted Developmental Surveillance Rates by State
