# Supplementary material for: Prevalence and Variation of Developmental Screening and Surveillance in Early Childhood
Source: JAMA Pediatr. 2018 Jul 9;172(9):857–66. doi: 10.1001/jamapediatrics.2018.1524 (PMC6143066; doi:10.1001/jamapediatrics.2018.1524)
Supplement: Supplement. — eTable 1. Adjusted Associations Between Medical Home Components and Developmental Screening and Surveillance eTable 2. Adjusted Associations With Developmental Screening and Surveillance (Without Preventive Medical Visit and Special Health Care Needs Status) eTable 3. Unadjusted and Adjusted Developmental Screening Rates by State eTable 4. Unadjusted and Adjusted Developmental Surveillance Rates by State [file jamapediatr-172-857-s001.pdf]

Supplementary Online Content

Hirai AH, Kogan MD, Kandasamy V, Reuland C, Bethell C. Prevalence and variation of developmental screening and surveillance in early childhood. *JAMA Pediatr.* Published online July 9, 2018. doi:10.1001/jamapediatrics.2018.1524

eTable 1. Adjusted Associations Between Medical Home Components and Developmental Screening and Surveillance

eTable 2. Adjusted Associations With Developmental Screening and Surveillance (Without Preventive Medical Visit and Special Health Care Needs Status)

eTable 3. Unadjusted and Adjusted Developmental Screening Rates by State

eTable 4. Unadjusted and Adjusted Developmental Surveillance Rates by State

This supplementary material has been provided by the authors to give readers additional information about their work.

© 2018 Hirai et al. *JAMA Pediatrics.*

eTable 1. Adjusted Associations Between Medical Home Components and Developmental Screening and Surveillance[a]

	Developmental Screening n=5,129			Developmental Surveillance n=5,269		
**Medical Home Component**	**Adjusted Prevalence**	**Rate Ratio**	**(95% CI)**	**Adjusted Prevalence**	**Rate Ratio**	**(95% CI)**
**Usual Source of Care**						
Yes	33.7	2.14	(1.55 - 2.96)	38.4	1.29	(0.99 - 1.68)
No	15.7	REF		29.8	REF	
**Personal Doctor or Nurse**						
Yes	33.1	1.26	(1.03 - 1.53)	39.0	1.24	(1.04 - 1.48)
No	26.3	REF		31.4	REF	
**Family-Centered Care**						
Yes	33.3	1.62	(1.18 - 2.24)	38.5	1.28	(0.99 - 1.65)
No	20.5	REF		30.0	REF	
**Referrals if needed**						
Yes	37.2	1.08	(0.73 - 1.60)	40.7	0.93	(0.66 - 1.30)
No	34.3	REF		43.9	REF	
Not needed	30.2	0.88	(0.60 - 1.29)	36.1	0.82	(0.60 - 1.13)
**Care Coordination if needed**						
Yes	33.2	0.94	(0.73 - 1.21)	37.9	1.07	(0.86 - 1.33)
No	35.4	REF		35.4	REF	
Not needed	29.7	0.84	(0.64 - 1.10)	37.2	1.05	(0.84 - 1.31)

[a]Adjusted for child age, sex, race/ethnicity, primary household language, family structure, highest household education, household income-to-poverty ratio, insurance coverage and type, preventive visit in past year, child health status, and special health care needs status.

© 2018 Hirai et al. *JAMA Pediatrics.*

eTable 2. Adjusted Associations With Developmental Screening and Surveillance (Without Preventive Medical Visit and Special Health Care Needs Status)[a]

	Developmental Screening n=5,268			Developmental Surveillance n=5,416		
	Adjusted Prevalence	Rate Ratio	(95% CI)	Adjusted Prevalence	Rate Ratio	(95% CI)
Predisposing Characteristics						
**Age**						
9-23 months	29.9	REF		36.0	REF	
24-35 months	33.0	1.10	(0.95 - 1.28)	37.9	1.05	(0.93 - 1.20)
**Sex**						
Male	32.7	REF		38.4	REF	
Female	29.8	0.91	(0.79 - 1.05)	35.3	0.92	(0.81 - 1.05)
**Race/Ethnicity**						
Non-Hispanic White	31.0	REF		36.7	REF	
Non-Hispanic Black	31.1	1.00	(0.71 - 1.41)	33.1	0.90	(0.68 - 1.20)
Hispanic	31.8	1.02	(0.81 - 1.30)	36.8	1.00	(0.80 - 1.25)
Non-Hispanic Other Single Race	25.9	0.84	(0.59 - 1.19)	38.1	1.04	(0.77 - 1.39)
Non-Hispanic Multiple Race	36.9	1.19	(0.93 - 1.53)	44.1	1.20	(0.98 - 1.48)
**Primary Household Language**						
English	33.0	REF		38.3	REF	
Non-English	18.4	0.56	(0.36 - 0.88)	27.2	0.71	(0.49 - 1.03)
**Family Structure**						
Two Parent, Married	33.2	REF		38.7	REF	
Two Parent, Unmarried	30.5	0.92	(0.69 - 1.23)	36.9	0.95	(0.74 - 1.22)
Single Mother/Other	24.0	0.72	(0.54 - 0.97)	30.1	0.78	(0.61 - 0.99)
**Highest Household Education**						
Less than high school	27.8	0.79	(0.43 - 1.46)	25.6	0.65	(0.35 - 1.18)
High school	25.1	0.72	(0.54 - 0.96)	31.7	0.80	(0.63 - 1.02)
Some college	26.6	0.76	(0.61 - 0.94)	36.8	0.93	(0.78 - 1.11)
College degree or higher	35.0	REF		39.7	REF	
Enabling Characteristics						
**Household Income-to-Poverty Ratio**						
<100% Poverty	33.9	1.18	(0.80 - 1.73)	37.5	1.07	(0.82 - 1.40)
100-199% Poverty	31.2	1.09	(0.82 - 1.44)	43.1	1.23	(0.98 - 1.53)
200-399% Poverty	32.9	1.14	(0.95 - 1.38)	34.3	0.98	(0.83 - 1.15)
400%+ Poverty	28.8	REF		35.1	REF	
**Insurance Coverage and Type**						
Any Public	30.5	0.95	(0.75 - 1.20)	40.0	1.12	(0.91 - 1.38)
Private only	32.1	REF		35.7	REF	
Uninsured	23.7	0.74	(0.42 - 1.28)	29.8	0.84	(0.48 - 1.47)

© 2018 Hirai et al. *JAMA Pediatrics.*

	Developmental Screening n=5,268				Developmental Surveillance n=5,416			
	Adjusted Prevalence	Rate Ratio	(95% CI)		Adjusted Prevalence	Rate Ratio	(95% CI)	
**Medical Home**								
Has Medical Home	35.0	1.33	(1.13 -	1.57)	40.1	1.22	(1.07 -	1.40)
No Medical Home	26.3	REF			32.8	REF		
Need Characteristics								
**Child Health Status**								
Excellent/Very Good	31.8	REF			36.4	REF		
Good/Fair/Poor	22.7	0.71	(0.50 -	1.03)	45.0	1.24	(0.96 -	1.59)

[a]Preventive visit in past year and special health care needs status are removed from the model out of concern for potential mediation and reverse causality, respectively.

© 2018 Hirai et al. *JAMA Pediatrics.*

eTable 3. Unadjusted and Adjusted Developmental Screening Rates by State

State	Unadjusted Estimate	Unadjusted (95% CI)	Adjusted Estimate	Adjusted (95% CI)
US	30.4	(28.0 -33.0)	30.4	(28.0 -33.0)
AL	21.2	(13.1 -32.3)	24.0	(16.0 -34.5)
AK	**46.8**	**(35.1 -58.8)**	**45.7**	**(34.6 -57.3)**
AZ	30.8	(18.9 -46.0)	32.8	(19.6 -49.5)
AR	20.7	(12.1 -33.0)	**18.4**	**(10.8 -29.5)**
CA	22.4	(12.8 -36.3)	26.6	(16.2 -40.4)
CO	**50.2**	**(37.8 -62.6)**	**53.7**	**(42.0 -64.9)**
CT	28.3	(18.2 -41.2)	27.8	(18.1 -40.1)
DE	26.9	(18.3 -37.6)	23.6	(15.6 -34.1)
DC	29.3	(20.4 -40.1)	31.1	(21.6 -42.5)
FL	**20.4**	**(12.8 -30.9)**	22.4	(14.3 -33.3)
GA	37.1	(24.7 -51.4)	39.2	(27.4 -52.4)
HI	32.0	(22.9 -42.6)	31.0	(22.1 -41.7)
ID	24.5	(15.8 -35.8)	**19.6**	**(12.6 -29.2)**
IL	39.4	(28.1 -52.0)	39.8	(28.4 -52.4)
IN	28.7	(19.5 -40.1)	31.0	(20.7 -43.5)
IA	34.8	(25.4 -45.7)	32.5	(22.4 -44.5)
KS	41.6	(30.1 -54.2)	40.3	(28.9 -52.8)
KY	**17.5**	**(10.6 -27.5)**	**17.6**	**(10.6 -27.9)**
LA	21.1	(12.1 -34.0)	**18.1**	**(10.2 -30.0)**
ME	31.6	(21.4 -44.0)	33.2	(21.4 -47.4)
MD	**43.0**	**(31.4 -55.6)**	39.3	(28.4 -51.3)
MA	**46.3**	**(35.2 -57.7)**	41.7	(30.4 -53.9)
MI	26.8	(18.5 -37.1)	26.1	(18.5 -35.4)
MN	**50.1**	**(39.5 -60.8)**	**49.8**	**(39.9 -59.7)**
MS	17.2	(6.6 -37.6)	20.2	(7.6 -44.0)
MO	23.4	(15.6 -33.6)	22.7	(15.5 -31.9)
MT	**46.3**	**(34.7 -58.4)**	39.0	(28.4 -50.8)
NE	33.1	(24.3 -43.4)	31.5	(22.8 -41.7)
NV	30.9	(18.4 -47.0)	27.2	(16.5 -41.4)
NH	32.0	(21.4 -44.9)	32.8	(21.9 -45.9)
NJ	32.9	(21.6 -46.6)	32.2	(20.5 -46.7)
NM	38.4	(24.1 -54.9)	42.6	(27.3 -59.4)
NY	**17.5**	**(9.9 -29.1)**	23.4	(14.9 -34.8)
NC	**47.6**	**(34.3 -61.3)**	**46.8**	**(33.3 -60.8)**
ND	30.0	(21.9 -39.6)	27.3	(19.6 -36.7)
OH	41.1	(29.6 -53.6)	40.7	(28.6 -54.1)
OK	35.1	(23.5 -48.8)	36.9	(24.8 -50.9)
OR	**58.8**	**(46.0 -70.5)**	**59.8**	**(47.5 -71.0)**
PA	26.7	(17.5 -38.6)	26.0	(17.4 -36.9)
RI	34.1	(23.5 -46.6)	33.3	(24.0 -44.2)

© 2018 Hirai et al. *JAMA Pediatrics.*

State	Unadjusted Estimate	(95% CI)	Adjusted Estimate	(95% CI)
SC	20.6	(12.1 -33.0)	21.4	(12.5 -34.3)
SD	40.4	(30.2 -51.5)	34.8	(25.1 -45.9)
TN	26.2	(17.0 -38.1)	26.5	(17.0 -39.0)
TX	34.1	(22.8 -47.5)	36.4	(26.5 -47.7)
UT	33.1	(24.3 -43.4)	29.1	(21.0 -38.9)
VT	39.7	(28.2 -52.6)	35.3	(24.5 -47.8)
VA	26.8	(17.5 -38.7)	26.5	(17.2 -38.5)
WA	31.9	(21.6 -44.3)	25.8	(17.0 -37.0)
WV	33.2	(21.1 -48.1)	32.6	(20.7 -47.3)
WI	25.9	(17.7 -36.2)	23.5	(16.4 -32.3)
WY	27.6	(18.4 -39.1)	25.3	(16.5 -36.8)
Variance	89.2		85.6	

Bold = significantly different from US, p<0.05
[a]Adjusted for child age, sex, race/ethnicity, primary household language, family structure, highest household education, household income-to-poverty ratio, insurance coverage and type, medical home, preventive visit in past year, child health status, and special health care needs status.

© 2018 Hirai et al. *JAMA Pediatrics.*

eTable 4. Unadjusted and Adjusted Developmental Surveillance Rates by State

State	Unadjusted Estimate	Unadjusted (95% CI)	Adjusted[a] Estimate	Adjusted[a] (95% CI)
US	37.1	(34.4 -39.8)	37.1	(34.4 -39.8)
AL	29.2	(18.4 -43.1)	31.7	(20.2 -46.0)
AK	**53.9**	**(41.8 -65.5)**	**54.2**	**(41.8 -66.1)**
AZ	36.5	(23.9 -51.3)	36.5	(23.4 -52.0)
AR	44.2	(30.9 -58.4)	41.1	(27.3 -56.5)
CA	27.9	(16.8 -42.5)	**25.8**	**(16.2 -38.4)**
CO	44.8	(33.0 -57.2)	46.8	(32.8 -61.3)
CT	47.7	(35.0 -60.6)	48.3	(35.7 -61.2)
DE	40.4	(28.8 -53.2)	39.4	(27.7 -52.5)
DC	37.3	(27.3 -48.4)	39.3	(29.3 -50.2)
FL	37.0	(25.1 -50.6)	36.6	(25.9 -48.8)
GA	33.7	(21.8 -48.2)	34.6	(22.6 -49.0)
HI	36.7	(27.1 -47.5)	36.5	(26.3 -48.0)
ID	**49.8**	**(38.9 -60.8)**	43.2	(33.2 -53.8)
IL	42.2	(31.0 -54.2)	43.8	(33.1 -55.0)
IN	32.5	(22.4 -44.5)	31.4	(21.6 -43.2)
IA	**59.4**	**(48.6 -69.4)**	**55.5**	**(44.9 -65.7)**
KS	31.5	(22.1 -42.6)	32.3	(23.2 -43.0)
KY	28.8	(19.0 -41.2)	29.9	(19.3 -43.1)
LA	31.2	(19.0 -46.6)	25.4	(15.3 -39.0)
ME	41.5	(30.0 -53.9)	39.8	(27.8 -53.1)
MD	47.3	(35.1 -59.8)	43.3	(31.3 -56.1)
MA	**54.9**	**(43.5 -65.9)**	48.3	(37.1 -59.7)
MI	34.1	(24.3 -45.5)	36.1	(26.4 -47.2)
MN	45.8	(35.4 -56.6)	47.0	(36.6 -57.7)
MS	**19.1**	**(9.0 -36.0)**	**21.0**	**(9.8 -39.5)**
MO	38.1	(26.8 -50.8)	38.3	(26.8 -51.2)
MT	42.1	(31.2 -53.9)	37.5	(27.3 -48.9)
NE	32.8	(23.9 -43.1)	32.7	(23.7 -43.2)
NV	33.4	(22.7 -46.2)	35.6	(24.0 -49.2)
NH	30.6	(21.4 -41.6)	30.1	(21.1 -40.8)
NJ	26.7	(16.8 -39.7)	26.9	(17.5 -39.0)
NM	36.8	(23.6 -52.4)	30.6	(17.9 -47.1)
NY	32.5	(21.4 -45.9)	39.2	(25.6 -54.7)
NC	49.5	(36.3 -62.9)	47.2	(34.4 -60.3)
ND	40.4	(31.5 -50.0)	38.4	(29.5 -48.1)
OH	40.3	(29.0 -52.7)	38.2	(26.5 -51.4)
OK	30.6	(20.5 -43.1)	30.4	(20.3 -42.9)
OR	**60.8**	**(49.0 -71.5)**	**61.1**	**(49.4 -71.7)**
PA	36.6	(25.8 -49.0)	37.2	(27.0 -48.7)
RI	42.6	(31.1 -55.0)	40.0	(28.7 -52.4)

© 2018 Hirai et al. *JAMA Pediatrics.*

State	Unadjusted Estimate	Unadjusted (95% CI)	Adjusted[a] Estimate	Adjusted[a] (95% CI)
SC	41.7	(29.2 -55.3)	41.1	(29.1 -54.3)
SD	44.4	(34.4 -54.9)	42.5	(32.2 -53.4)
TN	42.5	(30.9 -55.0)	41.1	(29.6 -53.5)
TX	30.9	(19.9 -44.6)	31.4	(21.7 -43.1)
UT	47.3	(37.3 -57.5)	45.9	(35.9 -56.3)
VT	39.4	(27.9 -52.2)	33.9	(23.3 -46.5)
VA	43.3	(32.2 -55.1)	38.8	(28.4 -50.3)
WA	47.1	(35.0 -59.5)	42.9	(31.1 -55.6)
WV	48.5	(34.8 -62.5)	51.2	(37.2 -65.0)
WI	39.0	(28.8 -50.2)	37.5	(27.8 -48.4)
WY	41.8	(30.3 -54.4)	38.9	(27.0 -52.3)
Variance	70.5		61.5	

Bold = significantly different from US, p<0.05
[a]Adjusted for child age, sex, race/ethnicity, primary household language, family structure, highest household education, household income-to-poverty ratio, insurance coverage and type, medical home, preventive visit in past year, child health status, and special health care needs status.

© 2018 Hirai et al. *JAMA Pediatrics.*
